# Amalgam

**Published:** 1856-12

**Authors:** J. D. White


### AMALGAM.

BY J. D. WHITE.

We referred to this substance for filling teeth, in the News Letter some time since, and we did not expect at that time ever to have occasion to do so again; but, "it is somehow held to be more creditable to learn what one has not known, than to unlearn what he has erroneously believed." We were told by the author of this language that amalgam as he recommended it, or the new method of preparing it for use, rendered it free "from all the objections which had been urged against it by its opposers." We most candidly "*believed*," from our acquaintance with and confidence in our author, that it was free from at least some of the objections urged against it. But, we have lived long enough to "*unlearn*" what we "*erroneously believed!*" We would be glad if those who use amalgam would report their failures; if so, we should forever remain silent on the matter; but, in the language of an intelligent patient to us, "It is your bounden duty to make my case known to the public." We cannot find fault with young practitioners for resorting to this substance for filling large cavities, in order to retain their patients in their hands; yet, we believe that it would be more to their credit to send their patients to older and more competent operators for such *capital operations*, as is the case among young surgeons, until, by simple operations, their skill would be developed into that maturity which would enable them to perform that class of operations themselves. It cannot be denied that there are many operations of plugging which cannot be well performed by a very young or a very old practitioner, but only by one who is in full possession of his faculties, and in the prime of life. A proper understanding in this direction, in an enlightened profession, would better preserve its character before the world than a resort by a few, to expedients which are liable frequently to end in disgrace to the whole profession. The following cases will, doubtless, explain the remarks that we have made:—

Case I.—A gentleman called on us, from a young dentist, to have his teeth examined, as they had become uncomfortable and very much blackened. We found four large cavities plugged with amalgam of the new kind; they had been done about six months. The dentist assured the patient that the teeth could not be plugged with gold. We found no difficulty in plugging the teeth with gold.

Case II.—A young lady consulted us to know why her teeth looked so dark. We found three of them plugged with amalgam; they had been plugged about three weeks. One was a left inferior bicuspid; front part and crown surface decayed, and communicating. The patient inquired of her dentist, at the time, why he did not plug it with gold. He answered that it could not be done; the cavity was *too large*. The others were both right inferior bicuspids, approximal surfaces—both small cavities. When the patient inquired why these were not plugged with gold, he answered that the cavities were not *well shaped.* This dentist was old enough and competent to have plugged them well with gold, as many good plugs in the mouth testified, and had been done years ago.

Case III.—A lady from a distance had a front tooth plugged with the new amalgum; the dentist recommended it on the faith of what had been said about it in this journal. He assured the patient that it would not discolor. The patient found the tooth turning blue; and, before she could see her dentist, it was quite black. When she saw her dentist, he told her that the tooth was ruined, and must be cut off and another set on. The patient, however, preferred to employ another dentist rather than submit to this operation by him.

Case IV.—An elderly lady had a left inferior first bicuspid plugged; nerve dead. She called to inquire whether it caused her mouth to be sore, and if so, she wished it extracted, as it made her "spit all the time." The tooth was extracted. It was new amalgum; had been in the tooth three months. She inquired, "Why do people undertake to fix teeth who do not understand it?" The exposed surface of the plug was very bright, but the tooth as black as ink. This has been true of many cases that we have seen; the surfaces of the plugs were bright, but the teeth black beneath them.

Case V.—Mr. —— S., 44 years of age, generally in good health, bilious-nervous temperament, had a large molar plugged with amalgam. His health became bad. He could not sleep at night, as the water accumulated so much in his mouth. He consulted his doctor, and he told him he was suffering from salivation. He called on his dentist, and told him how he had been affected since he had plugged his tooth. The dentist at once exclaimed, "Why, you are salivated! There has been a *little free mercury left in the plug.* I will take it out, and put one in that will be free from such objection. It is a new thing, and contains no free mercury." The plug was put in, but it turned black also; and in about two months the same set of symptoms set in; constant spitting, day and night, accompanied with stricture across the chest and about the throat; frequent spots of ulceration of the mucous membrane of the mouth, and excoriations of the corners of the mouth; debility and emaciation, with loss of appetite, supervened. The sputa was frequently white and frothy. He had fever of the mouth, and heavy breath, and sometimes oppression. The patient remarked that he would not go through with the same thing that he had done the last month, before the plug was removed, for five hundred dollars, and that it was our bounden duty to communicate it to the world, for the benefit of mankind.—*Ib.*

